# Epidemiological characteristics and co-occurrence patterns of *Rothia* species and respiratory pathogens: from population surveillance to mechanistic insights

**DOI:** 10.1080/20002297.2026.2657114

**Published:** 2026-04-14

**Authors:** Zhen Wang, Yujie Fang, Jing Li, Jing Li, Xingru Zhu, Ruyu Tian, Keying Chen, Yan Xiao, Lixia Zhang, Jie Liu

**Affiliations:** a School of Public Health, Qingdao University, Qingdao, Shandong, China; b Department of Clinical Laboratory, The Affiliated Hospital of Qingdao University, Qingdao, Shandong, China; c Department of Medicine, University of North Carolina at Chapel Hill School of Medicine, Chapel Hill, NC, USA

**Keywords:** *Rothia*, respiratory pathogens, co-infection, pneumonia, influenza A virus, *Klebsiella pneumoniae*, quantitative polymerase chain reaction

## Abstract

**Background:**

*Rothia* is an oral commensal bacterium increasingly detected in the respiratory tract, and recent studies have highlighted changes in its abundance in respiratory infections.

**Objective:**

This study aimed to investigate the species distribution of *Rothia*, its co-occurrence with respiratory pathogens, and the immune alterations associated with *Rothia* co-infection in respiratory infections.

**Design:**

We developed real-time PCR to differentiate *R. aeria, R. dentocariosa,* and *R. mucilaginosa*. Sputum samples from 447 hospitalized patients were tested for 35 respiratory pathogens and three *Rothia* species using customized TaqMan Array Card. Co-infection murine models were established.

**Results:**

All three *Rothia* species were frequently detected (39.4% to 72.3%). *R. mucilaginosa* showed positive associations with SARS-CoV-2 among patients with respiratory symptoms (odds ratio 4.0, *p* < 0.0001) or pneumonia (OR 2.7, *p* = 0.019) and influenza A virus (IAV; OR 8.4, *p* = 0.004) in patients with pneumonia during an influenza season, but was negatively associated with *Klebsiella pneumoniae* (OR 0.3, *p* < 0.05). These inverse associations were confirmed in murine models. Increased mortality and bacterial burden were observed in IAV and *R. mucilaginosa* co-infection, while *K. pneumoniae* and *R. mucilaginosa* co-infection was attenuated. Both models showed increased neutrophils. Functionally, IAV co-infection impaired bactericidal activity whereas prior *K. pneumoniae* exposure enhanced bacterial clearance.

**Conclusions:**

This study uncovers distinct pathogen associations of *Rothia* in respiratory infections, which may provide evidence for their roles in shaping disease outcomes.

## Article highlights

- Simultaneous detection of *Rothia* species and respiratory pathogens in hospitalized patients with a syndromic approach revealed the distinct associations of *Rothia,* i.e. positively with SARS-CoV-2 and influenza while negatively with *Klebsiella pneumoniae*.
- Murine co-infection models confirmed inverse associations of *Rothia* with pathogens, demonstrating increased mortality and bacterial burden in influenza and *R. mucilaginosa* co-infection, in contrast to attenuated *K. pneumoniae* and *R. mucilaginosa* co-infection.
- Both models showed increased neutrophils but difference in bactericidal activity, indicating divergent infection progression and outcomes may be attributed to altered neutrophil function.

## Graphical abstract

**Figure:**
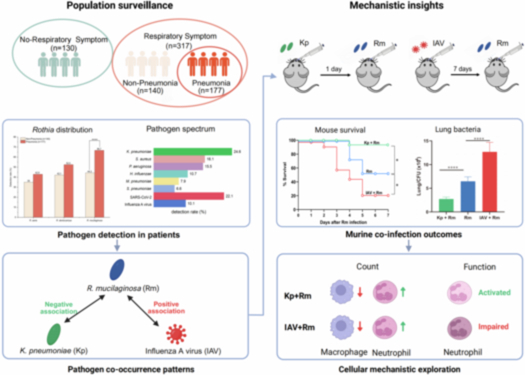

## Introduction

*Rothia* species are Gram-positive commensals colonising the human oral and respiratory tracts [1]. While widely regarded as commensals, *Rothia* has also been reported in systemic infections such as endocarditis and septicaemia [2]. Of the 11 currently recognised species, *Rothia mucilaginosa*, *Rothia dentocariosa*, and *Rothia aeria* are most frequently pathogenic in humans [3].

Yet evidence indicates that *Rothia* engages in diverse interactions with other pathogens, including inhibiting *Moraxella catarrhalis* [4], *Streptococcus mutans* [5], and *Staphylococcus aureus*, co-occurring with *Porphyromonas gingivalis* and *Fusobacterium nucleatum* [6]. *Rothia* has also been found enriched in the respiratory microbiota during infections with Severe Acute Respiratory Syndrome Coronavirus 2 (SARS-CoV-2) [7,8], especially in those with more severe respiratory symptoms [9], and in patients with influenza virus [10]. Controversially, it was reported that *Rothia* may decrease in abundance in COVID-19 patients [11,12], indicating complicated interactions or unknown confounding factors. As these studies were mostly based on metagenomic sequencing of clinical specimens from patients with one specific infection, their ecological roles in respiratory infections, particularly in the broad context of pathogen association, remain inconclusive and lack experimental validation.

Nonetheless, *Rothia* is not commonly targeted in clinical microbiology. Accurate identification of *Rothia* is complicated by its phenotypic similarity to *Actinomyces*, *Corynebacterium*, and *Nocardia* [13]. Therefore, culture-based methods often misidentify isolates. Special medium such as oral *Rothia* species selective medium (ORSM) and a limited number of molecular assays were developed [14,15], but have not been widely utilised. The identification of *Rothia* in complex clinical specimens has mostly relied on metagenomic sequencing [7,8,10,11]. Practical and scalable molecular diagnostics capable of rapid, quantitative, and species-specific detection of *Rothia* are still lacking, which hampers investigations into its role in respiratory infections.

Accordingly, we aimed to: (i) develop and validate qPCR assays for three major *Rothia* species, which are made compatible with the TaqMan Array Card (TAC), a microfluidic qPCR based platform; (ii) characterise *Rothia* co-occurrence patterns with respiratory pathogens in a clinical setting; and (iii) use murine co-infection models to verify clinical findings and assess alterations in cell function.

## Materials and methods

### Study population and case definition

Hospitalised patients were enroled at the Affiliated Hospital of Qingdao University between May 2023 and April 2025 (Figure S1). The enrolment criteria included patients with sputum collected within 48 hours of admission and with complete records. We excluded individuals with tuberculosis, lung cancer, or non-infectious interstitial lung disease. Patients were assigned to the respiratory symptom group if they presented with at least one respiratory feature and one systemic feature. Those without any of these features comprised the no respiratory symptom group.

(1) Respiratory features: new or worsening cough, sputum, purulence, chest pain, or compatible auscultatory findings.
(2) Systemic features: fever (axillary temperature ≥ 37.5°C), chills, malaise, fatigue, or myalgias.

Patients in the respiratory symptom group were further subdivided into pneumonia or non-pneumonia groups based on the presence of new radiographic pulmonary infiltrates according to established guidelines [16].

To evaluate the representativeness of hospitalised control group for *Rothia* carriage, a convenient set of healthy participants were enroled from the community, with basic demographic and health information collected.

This study was approved by the ethics committees of Qingdao Medical College of Qingdao University (QDU-HEC-2024246) and conducted in accordance with the Declaration of Helsinki. Informed consent was obtained from all participants upon admission.

### Specimen collection and nucleic acid extraction

Sputum specimens were collected following standard protocol and stored at −80 °C. Reference strains *R. mucilaginosa* ATCC 25296 and *R. dentocariosa* ATCC 17931 purchased from Shanghai Microbiological Culture Collection Co. Ltd. (CHMCC), and a plasmid (pUC57) containing the target fragment of *R. aeria* (Tsingke Biological Technology, Beijing, China) were used as positive controls in assay development and validation.

Total nucleic acids were extracted from sputum using an automated QIAcube HT (QIAGEN, Hilden, Germany) according to the manufacturer’s protocol. Briefly, sputum samples (200 µL) were mixed with an equal volume of 0.2% dithiothreitol (DTT) and incubated on a shaker for 3 h on ice, followed by the addition of 800 µL InhibitEX buffer (QIAGEN, Germany) and homogenisation. A 600 µL aliquot of the homogenised sputum was transferred to the sample plate for automated nucleic acid extraction on a QIAcube HT instrument (QIAGEN, Hilden, Germany) using the Pathogen 96 Kit (QIAGEN). External controls, Phocine herpesvirus and MS2 bacteriophage were spiked into each sample, and an extraction blank was included in each batch as a quality control.

### qPCR design and setup

Primers and probes for 3-plex *Rothia* qPCR were designed from conserved genomic regions and synthesised commercially (Table 1). *In silico* specificity was assessed using BLAST analysis against the NCBI database. The 3-plex qPCR was performed on a QuantStudio™ 7 Flex Real-Time PCR System (ThermoFisher, Carlsbad, CA, USA) with primer and probe concentrations listed in Table 1. Reactions were set up in 10-μL volumes using TaqMan™ Fast Virus 1-Step Master Mix. The reaction conditions were: reverse transcription at 50 °C for 5 min, initial denaturation at 95 °C for 20 s, and 40 cycles of 95 °C for 3 s and 60 °C for 30 s. A quantification cycle (Cq) cut-off of 35 was applied for positivity. Each run included the positive and no-template controls.

**Table 1.** Design and validation of the *Rothia* multiplex qPCR panel.

Target species	Sequence (5'→3')	Conc (nM)	PCR efficiency (%)	Linearity R^2^	Precision (%)		LoD^a^ (CFU/μL)
					Intra-assay	Inter-assay	
*R. aeria*	F: CCGACCATTTCTGCACCA	240	102·6	0·995	2·4	2·4	38
	R: AGGCCCATCATTGGCTAATAC	240					
	P: TAMRA-CAAACCGGCCCAAACCGATCAC	120					
*R. dentocariosa*	F: CTTTGTTGTGGGCGGATTATG	120	98·5	0·991	2·0	3·6	45
	R: CGCCGCTATCATTCCTATCA	120					
	P: VIC-CCGCATAGCCTTCGGACCGTTTAT	60					
*R. mucilaginosa*	F: GCATCCATTGCGGCATATAAC	120	97·4	0·996	1·7	2·2	59
	R: CAAAGGTCGACGGAGCAATA	120					
	P: FAM-CCAATGAAGGTAGCGAGCAGCTGA	60					

Amplicons locate at nt 886,348–886,496 (*R. aeria*, AP017895.1; potassium uptake protein TrkA), nt 1,514,384–1,514,492 (*R. dentocariosa*, CP002280.1; A24 family peptidase), and nt 1,774,558–1,774,693 (*R. mucilaginosa*, AP014938.1; lipolytic enzyme).​​​​

^a^ LoD, defined as the lowest concentration in sputum samples yielding 100% detection across 20 replicates.

### Validation of *Rothia* qPCR

The analytical performance of the 3-plex *Rothia* qPCR panel was evaluated for linearity, PCR efficiency, precision, limit of detection (LoD), and specificity following established guidelines [17]. Amplification efficiency and linearity were evaluated using standard curves generated from serial dilutions of genomic DNA from *Rothia* strains and the synthetic construct for *R. aeria,* each tested in triplicate. Precision (intra- and inter-assay) was determined from 20 replicates at high and low concentrations. Results were expressed as coefficients of variation (CV). The LoD was defined as the lowest concentration at which all 20 replicates were tested positive. Analytical specificity was evaluated by performing the *Rothia* 3-plex qPCR panel on non-target bacterial genomic DNA. For confirmation, randomly selected *Rothia*-positive sputum samples were retested by conventional PCR generating longer amplicons followed by amplicon sequencing (Tables S1 and S2).

### Pathogen detection with TAC

Respiratory pathogens were detected with a customised TAC, allowing identification of 19 bacterial species, including three *Rothia* species, 19 viruses, and 24 antimicrobial resistance genes (Figure S2). The TAC procedures were performed as previously described [18], using the TaqMan™ Fast Virus 1-Step Master Mix under the same cycling conditions as the *Rothia* qPCR.

### Mouse model for lung infection

Female CD1 mice (6-8 weeks old) were maintained under specific pathogen-free conditions. All animal procedures were approved by the Laboratory Animal Centre of Qingdao University (20241206ICR10520250819206) and conducted in accordance with the institutional guidelines.

### Bacterial and viral cultivation

*R. mucilaginosa* ATCC 25296 was cultured in Brain Heart Infusion (BHI) broth (BD Biosciences), and *Klebsiella pneumoniae* ATCC 35657 was grown on Luria-Bertani (LB) medium using standard microbiological methods [1,19]. The mouse-adapted PR8 strain of influenza A virus (IAV) was propagated in 9-day-old embryonated chicken eggs and titrated by plaque assay on Madin-Darby canine kidney (MDCK) cells as previously described [20].

### Lung infection

Female CD1 mice (6–8 weeks old) were obtained from Vital River Laboratory Animal Co., Ltd. (Beijing, China). As described previously [21], under anaesthesia, mice were intranasally infected with *R. mucilaginosa* (10⁷ CFU), either alone or following infection with influenza A virus (30 PFU) or *K. pneumoniae* (10⁴ CFU). Survival was recorded with humane endpoints defined as > 20% weight loss. At designated time points, lungs were homogenised for quantitative culture via serial dilution.

### Histopathological analysis

Lung tissues were collected 12 hours after infection or co-infection, and mock-treated animals were sampled at the same time point, fixed in 4% paraformaldehyde, embedded in paraffin, sectioned, and stained with hematoxylin and eosin (H&E). Histopathological changes were evaluated by blinded semiquantitative scoring [22].

### Flow cytometry

Immune cell populations in bronchoalveolar lavage fluid (BALF) were analysed by flow cytometry as established procedures [23]. BALF was collected by flushing the lungs with PBS. After centrifugation and RBC lysis, approximately 5 × 10⁵ cells per sample were resuspended in FACS buffer and incubated with anti-CD16/32 antibody (BioLegend, San Diego, CA, USA). Cells were stained with PE-conjugated anti-SiglecF (BD Biosciences, San Jose, CA, USA) and FITC-conjugated anti-Ly6G (BioLegend, San Diego, CA, USA). Data were acquired on a CytoFLEX S flow cytometer (Beckman Coulter, Brea, CA, USA), and viable populations were gated as neutrophils (Ly6G⁺SiglecF⁻) and alveolar macrophages (SiglecF⁺Ly6G⁻). Total leucocyte counts were quantified using a TC20 automated cell counter (Bio-Rad Laboratories, Hercules, CA, USA).

### Bacterial killing assay

Lung phagocytes were isolated from mice at 12 hours post-challenge with *R. mucilaginosa*, following prior infection with either IAV or *K. pneumoniae*. Cells were isolated and treated with RBC lysis buffer (BioLegend, San Diego, CA, USA). Cells were seeded in serum-free RPMI 1640 medium (Corning, Corning, NY, USA) at 5 × 10^5^ cells/well. After adherence, cells were infected with *R. mucilaginosa* at a multiplicity of infection (MOI, bacterium vs cell) of 0.2 in 20% mouse serum, followed by centrifugation and a 1 hour incubation. As previously described [24], surviving bacteria were quantified by CFU enumeration after incubation with cells. Results were expressed as percentages relative to the inoculum.

### Transcriptomic analysis of sorted neutrophils

Neutrophils were isolated from mouse lung single-cell suspensions by sorting CD11b⁺Ly6G⁺ cells on a BD FACS Aria III (BD Biosciences, San Jose, CA, USA), and the post-sort purity was > 95%. Total RNA was extracted from neutrophils using TRIzol reagent (Invitrogen, Carlsbad, CA, USA). After RNA quality control, libraries were generated and subjected to transcriptome sequencing by Novogene (Beijing, China) on the Illumina NovaSeq X Plus system. Differential gene expression analysis was performed between the indicated groups. Functional enrichment analyses were then conducted using Gene Ontology (GO) [25], and Gene Set Enrichment Analysis (GSEA) [26] was performed to identify significantly enriched pathways.

### Statistical analysis

Categorical variables are presented as numbers (%) and compared using Chi-square or Fisher’s exact test. Continuous variables are presented as medians (IQR) and compared using the Mann–Whitney U test. Pairwise pathogen associations were assessed using univariate logistic regression and multivariable logistic regression adjusting for age, sex, and detection of key pathogens. Analyses were performed with SPSS version 26, and a two-sided *p* < 0.05 was considered significant.

## Results

### Development and validation of quantitative PCR for *Rothia* speciation

Through genomic sequence annotation, sequence alignment, and BLAST, we identified species-specific sequences in three *Rothia* species (Table 1). A TaqMan probe-based 3-plex real-time PCR panel was developed and validated with the analytical performance (Table 1). The assays demonstrated superior linearity (R² > 0.99) with amplification efficiency ranging from 97.4% to 102.6%. The lower limit of detection (LoD) in sputum was 38, 45, and 59 CFU/μL for *R. aeria, R. dentocariosa,* and *R. mucilaginosa*, respectively. All assays achieved high precision (CV < 5%) and showed no non-specific amplification (Table S1).

74 qPCR-positive sputum samples were randomly selected, and the target genes were amplified for Sanger sequencing (Table S2). BLAST analysis confirmed the *Rothia* speciation results with 100% concordance.

### Detection of *Rothia* species in sputum from hospitalised patients

Among 447 hospitalised patients (median age 63 years (IQR, 52–73); two-thirds female), patients with respiratory infections were mostly from the Pulmonary and Critical Care Medicine department and exhibited higher body temperature and heart rate than those without respiratory symptoms (*p* < 0.0001; Table 2). Within this group, pneumonia patients exhibited slightly elevated respiratory rates compared with non-pneumonia patients (*p* = 0.024).

**Table 2.** Demographic and clinical characteristics of all hospitalised patients.

	All	No respiratory symptoms	Respiratory symptoms	*p*-value	Respiratory symptoms		*p*-value
					Non-pneumonia	Pneumonia	
	(*N* = 447)	(*n* = 130)	(*n* = 317)		(*n* = 140)	(*n* = 177)	
**Age (years)**	63(52–73)	61(52–71)	65(52·5–74)	0·187	65·5(55–75)	63(51–74)	0·215
**Sex**							
Male	152(34·0%)	46(35·4%)	106(33·4%)	0·693	44(31·4%)	62(35·0%)	0·5
Female	295(66·0%)	84(64·6%)	211(66·6%)		96(68·6%)	115(65·0%)	
**Inpatient department**							
Pulmonary and critical care medicine	250(55·9%)	54(41·5%)	196(61·8%)	<0·001	89(63·6%)	107(60·5%)	0·57
Other department*	197(44·1%)	76(58·5%)	121(38·2%)		51(36·4%)	70(39·5%)	
**Vital signs**							
Temp (°C)	37·1(37–37·6)	36·9(36·5–37·1)	37·2(37·1–37·6)	<0·001	37·2(37·1–37·6)	37·2(37·1–37·6)	0·337
RR (min)	20(16–22)	20(16–22)	20(18–22)	0·827	20(16–22)	20(18–22)	0·024
HR (min)	80(70–100)	75(70–86)	83(72–110)	<0·001	80(70–110)	88(74–110)	0·303
DBP (mmHg)	75(70–90)	75(70–82·5)	75(70–90)	0·117	75(70–90)	75(70–90)	0·975
SBP (mmHg)	115(105–125)	105(105–125)	115(105–125)	0·071	115(105–125)	115(105–125)	0·884

Abbreviations: HR, heart rate; RR, respiratory rate; SBP, systolic blood pressure; DBP, diastolic blood pressure;

^*^ Other departments included Emergency Internal Medicine, Cardiac Surgery, Neurosurgery, among others; p-values from χ² exact tests or Mann–Whitney U tests.

**Figure 1.**
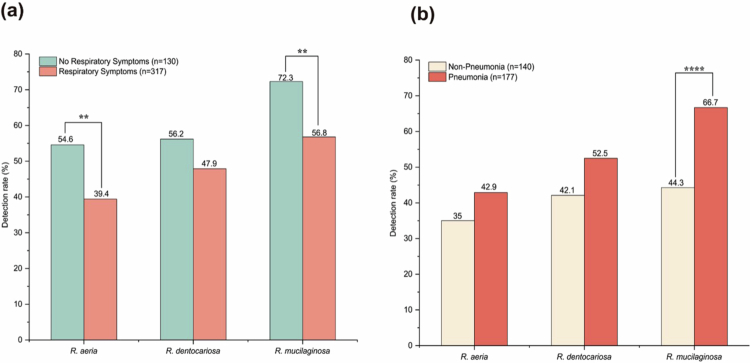
Prevalence of *Rothia* species in hospitalised patients from May 2023 to April 2025. (a) Detection rates of *Rothia* species in patients with (*n* = 317) and without (*n* = 130) respiratory symptoms. (b) Detection rates of *Rothia* species in patients with (*n* = 177) and without (*n* = 140) pneumonia. Chi-square test; **p < 0.01, ****p < 0.0001.

Of these 447 hospitalised patients, *Rothia* species were detected at strikingly high frequencies. In comparison between patients with and without respiratory infections (Figure 1a), significant differences were found in the detection rates of *R. aeria* (39.4% vs 54.6%; *p* = 0.003) and *R. mucilaginosa* (56.8% vs 72.3%; *p* = 0.002), while *R. dentocariosa* yielded similar detection rates (47.9% vs 56.2%; *p* = 0.12). In healthy community individuals, detection rates of all three *Rothia* species were even higher than those in hospitalised patients without respiratory symptoms (Table S3), exclusively >80%.

Within the 317 patients presenting with respiratory symptoms, the detection of *Rothia* species varied with pneumonia status (Figure 1b). The prevalence of *R. mucilaginosa* was significantly higher in pneumonia patients compared with those without pneumonia (66.7% vs 44.3%; *p* < 0.0001). In addition, both *R. aeria* (42.9% vs 35.0%; *p* = 0.15) and *R. dentocariosa* (52.5% vs 42.1%; *p* = 0.066) appeared more prevalent in pneumonia cases without statistical significance.

No significant difference was observed in quantities of the three *Rothia* species between the patients with and without respiratory infections (Cq 29.5, IQR 27.2–32.0 vs 28.9, 26.5–32.6; *p* = 0.42), or with and without pneumonia (30.2, 27.5–32.2 vs 28.8, 27.2–31.7; *p* = 0.081).

### Respiratory pathogen spectrum in hospitalised patients

We next characterised the spectrum of respiratory pathogens in sputum specimens from patients with respiratory symptoms (Table 3). Among bacterial pathogens, *Klebsiella pneumoniae* (24.6%) was the most frequently detected, followed by *Staphylococcus aureus* (16.1%), *Pseudomonas aeruginosa* (15.5%), and *Haemophilus influenzae* (10.7%). Less common detections included *Mycoplasma pneumoniae* (7.9%) and *Streptococcus pneumoniae* (6.6%).

**Table 3.** Prevalence of pathogens among patients stratified by respiratory symptoms and pneumonia status.

Pathogen	Respiratory symptoms	Respiratory symptoms	
		Non-pneumonia	Pneumonia
	(*n* = 317)	(*n* = 140)	(*n* = 177)
Bacteria			
*K. pneumoniae*	78 (24·6)	37 (26·4)	41 (23·2)
*S. aureus*	51 (16·1)	26 (18·6)	25 (14·1)
*P. aeruginosa*	49 (15·5)	25 (17·9)	24 (13·6)
*H. influenzae*	34 (10·7)	15 (10·7)	19 (10·7)
*M. pneumoniae*	25 (7·9)	2 (1·4)	23 (13·0)
*S. pneumoniae*	21 (6·6)	6 (4·3)	15 (8·5)
*M. catarrhalis*	9 (2·8)	5 (3·6)	4 (2·3)
*N. meningitidis*	8 (2·5)	5 (3·6)	3 (1·7)
*B. parapertussis*	8 (2·5)	3 (2·1)	5 (2·8)
*Legionella spp.*	3 (0·9)	1 (0·7)	2 (1·1)
*C. pneumoniae*	2 (0·6)	0 (0·0)	2 (1·1)
*M. tuberculosis*	2 (0·6)	1 (0·7)	1 (0·6)
Virus			
SARS-CoV-2	70 (22·1)	27 (19·3)	43 (24·3)
Influenza A virus	32 (10·1)	13 (9·3)	19 (10·7)
Human parainfluenza virus type 3	19 (6·0)	9 (6·4)	10 (5·6)
Human rhinovirus	15 (4·7)	4 (2·9)	11 (6·2)
Respiratory syncytial virus	9 (2·8)	5 (3·6)	4 (2·3)
Human coronavirus 229E	6 (1·9)	4 (2·9)	2 (1·1)
Human coronavirus OC43	6 (1·9)	2 (1·4)	4 (2·3)
Human metapneumovirus	4 (1·3)	1 (0·7)	3 (1·7)
Human adenovirus	3 (0·9)	1 (0·7)	2 (1·1)
Enterovirus	1 (0·3)	0 (0·0)	1 (0·6)
Human parainfluenza virus type 1	1 (0·3)	0 (0·0)	1 (0·6)
			

Pathogens with a detection rate >10% in the respiratory symptom group were defined as key pathogens and denoted by † in table; No positives were identified for the following targets: *P. jirovecii, S. pyogenes, C. psittaci, B. pertussis*; human parainfluenza virus type 2, human coronavirus HKU1, human coronavirus NL63, human bocavirus, influenza B virus, human parainfluenza virus type 4, SARS-CoV and Middle East respiratory syndrome coronavirus.

Among viral pathogens, SARS-CoV-2 (22.1%) and IAV (10.1%) predominated, especially in pneumonia cases. Other viruses, such as human parainfluenza virus type 3 (6.0%), human rhinovirus (4.7%), and respiratory syncytial virus (2.8%) were detected less frequently.

During the influenza season, the pathogen spectrum shifted, with increased detection of IAV (32.3%) and *M. pneumoniae* (10.8%) among patients with respiratory symptoms (Table S4).

### Co-occurrence of *Rothia* species with respiratory pathogens

**Figure 2.**
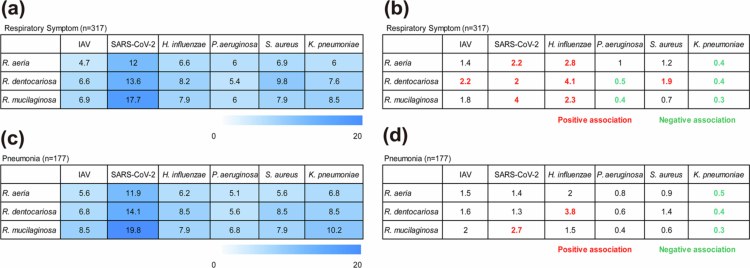
Pathogen co-occurrence and associations in patients with respiratory symptoms and the pneumonia subgroup. (a) and (c) Co-occurrence rates (%) for each pathogen pair. Darker colours indicate higher co-occurrence rates. (b) and (d) Pairwise associations shown as odds ratios (ORs). Colour indicates statistical significance (red, positive association; green, negative association; black indicates no significant association).

Among 317 patients with respiratory symptoms, *Rothia* species frequently co-occurred with major viral and bacterial pathogens (Figure 2a). *R. mucilaginosa* had the highest co-detection rate with SARS-CoV-2 (17.7%), followed by *R. dentocariosa* (13.6%) and *R. aeria* (12.0%). Logistic regression analysis confirmed these findings (Figure 2b) that all three *Rothia* species were positively associated with SARS-CoV-2 and *H. influenzae*, while negatively associated with *K. pneumoniae*. Although all three *Rothia* species showed a tendency to co-occur with IAV (ORs 1.4–2.2), these associations did not consistently reach statistical significance. Worth noting is that the positive association with *H. influenzae* disappeared when using a qPCR assay selective for encapsulated subtypes, which substantially reduced the detection rate of *H. influenzae* (data not shown).

In the pneumonia subgroup (*n* = 177), similar co-occurrence and association profiles were observed (Figure 2c, d). *R. mucilaginosa* remained significantly associated with SARS-CoV-2 (OR = 2.7, *p* = 0.019), while *R. dentocariosa* again correlated with *H. influenzae* (OR = 3.8, *p* = 0.015). Negative associations with *K. pneumoniae* persisted (ORs 0.3–0.5, *p* < 0.05). The corresponding detection rates and odds ratios with 95% CIs are summarised in Table S5.

*Rothia* Cq values were significantly lower in SARS-CoV-2 co-detection (28.1, 25.9–30.1 vs 30.0, 28.1–32.8 for *Rothia* alone; *p* = 0.005), whereas no difference was observed in *K. pneumoniae* co-detection (29.1, 28.0–31.0, *p* = 0.47).

### Prevalence and co-occurrence of *Rothia* in pneumonia patients during the influenza season

**Figure 3.**
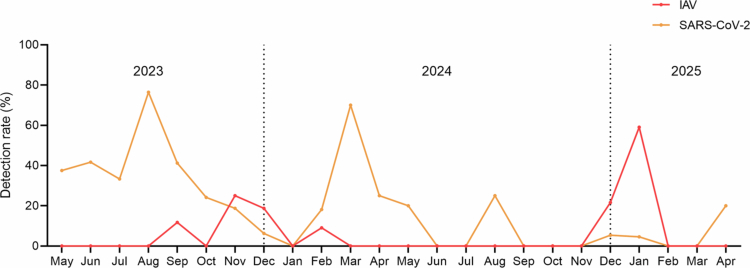
Temporal changes in IAV and SARS-CoV-2 detection in patients with respiratory symptoms from May 2023 to April 2025. The x-axis represents calendar months. Data are shown as percentage of positive cases among total tested patients per month.

We next examined the temporal dynamics of SARS-CoV-2 and IAV, the two major viral pathogens demonstrating co-occurrence with *Rothia* species, during the two-year study period. SARS-CoV-2 displayed multiple irregular peaks, with the highest detection in August 2023 (76.5%) and a smaller resurgence in April 2025 (Figure 3). In contrast, IAV activity was concentrated in the winter seasons, with an outbreak from December 2024 to February 2025, peaking at 59.1% in January, thereby offering an opportunity to further focus on the co-occurrence of *Rothia* and IAV.

**Figure 4.**
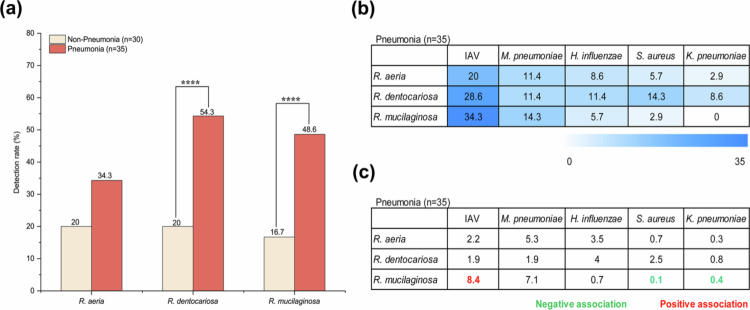
Prevalence and pathogen associations of *Rothia* during the 2024-2025 influenza season. (a) Detection of *Rothia* species in non-pneumonia and pneumonia patients. Chi-square test. (b) and (c) Co-occurrence rates (%) and pairwise pathogen associations, as in Figure 2.

During the influenza season spanning December 2024 to February 2025, both *R. dentocariosa* and *R. mucilaginosa* were significantly more prevalent in patients with pneumonia than in those without (*p* < 0.0001; Figure 4a). Co-occurrence analysis showed that *Rothia* species were frequently detected alongside IAV and *M. pneumoniae*, whereas overlap with *K. pneumoniae* was consistently rare (Figure 4b and Table S5b). Logistic regression further confirmed positive correlations of *R. mucilaginosa* with IAV (OR = 8.4, *p* = 0.004) and, marginally, with *M. pneumoniae* (OR = 7.1, *p* = 0.061), versus negative correlations with *S. aureus* and *K. pneumoniae* (Figure 4c). These observations highlight clinically relevant, pathogen-specific clustering of *Rothia* with respiratory pathogens, proposing that commensal-pathogen interactions might influence disease outcomes.

### Influence of pathogen exposure on subsequent *R. mucilaginosa* pulmonary infection in a murine model

**Figure 5.**
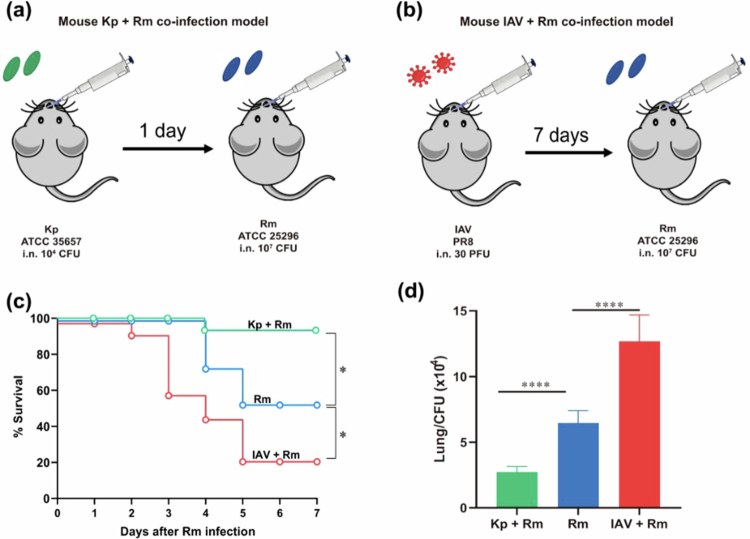
Effects of pathogen co-infection on *R. mucilaginosa* pulmonary infection. (a) and (b) experimental design of mouse co-infection models. ICR mice were first intranasally inoculated with *K. pneumoniae* or IAV, followed by *R. mucilaginosa*. (c) Survival curves of mice (*n* = 13–14 per group), analysed by log-rank test. (d) Lung bacterial burdens at 12 hour post-infection, determined by CFU counts (*n* = 6–7 per group). Mann–Whitney U test; ****p < 0.0001.

**Figure 6.**
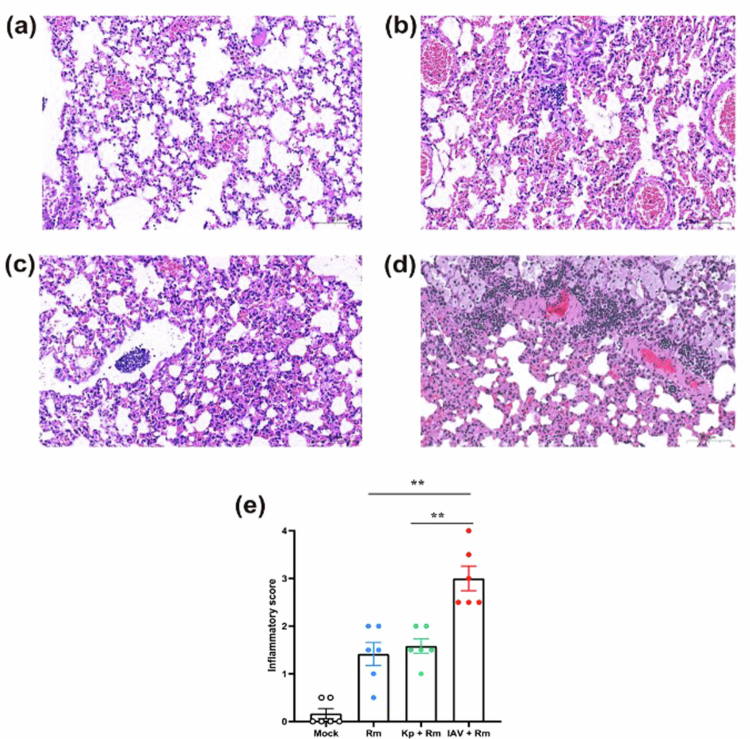
Histopathological evaluation of lung inflammation in murine models with *R. mucilaginosa* mono-infection and co-infection with IAV or *K. pneumoniae*. (a-d) Representative H&E-stained lung sections from the Mock, *R. mucilaginosa* mono-infection, *K. pneumoniae* and *R. mucilaginosa* co-infection, and IAV and *R. mucilaginosa* co-infection groups. Lung tissues were collected 12 hours after the final treatment. Scale bar, 100 μm. (e) Blinded semiquantitative inflammatory scores of lung sections (0–4). Higher scores indicate more severe inflammatory infiltration. Data are shown as individual values with median. Statistical analysis was performed using the Mann–Whitney U test. **p < 0.01.

Based on previous reports [27,28], the infection doses for IAV and *K. pneumoniae* were determined to be 30 PFU and 10⁴ CFU, respectively, that caused no mortality confirmed in the current study (Figure S3a). *R. mucilaginosa* exhibited dose-dependent lethality (Figure S3b), for which 10⁷ CFU was chosen as the infection dose, which produced a reproducible and measurable, but not fully saturating, infection phenotype, with approximately 40% mortality. Next, we established co-infection models (Figure 5a, b). Survival analysis revealed profoundly divergent outcomes between the two infection models. *R. mucilaginosa* mono-infection caused 40% mortality. Co-infection with *K. pneumoniae* significantly improved survival (16.7% mortality, *p* = 0.024), whereas IAV co-infection was highly fatal (78.6% mortality, *p* = 0.023; Figure 5c). At 12 hours post-challenge, pre-exposure to *K. pneumoniae* resulted in a 2.4-fold reduction in lung bacterial burden of *R. mucilaginosa* compared to mono-infection. Conversely, pre-exposure to IAV yielded a 2-fold increase in lung bacterial load (*p* < 0.0001; Figure 5d). Histopathological scoring further supported the divergent outcomes of the two co-infection models. Lung inflammatory injury was significantly more severe in the IAV and *R. mucilaginosa* co-infection group than in the *R. mucilaginosa* alone and *K. pneumoniae* and *R. mucilaginosa* co-infection groups (both *p* = 0.0065), whereas no significant difference was observed between the latter two groups (Figure 6e). This bidirectional modulation underscores the pivotal role of antecedent viral or bacterial exposure in shaping opposite innate immune responses against subsequent *Rothia* challenge.

### Cellular responses of leucocytes during pathogen co-infection

**Figure 7.**
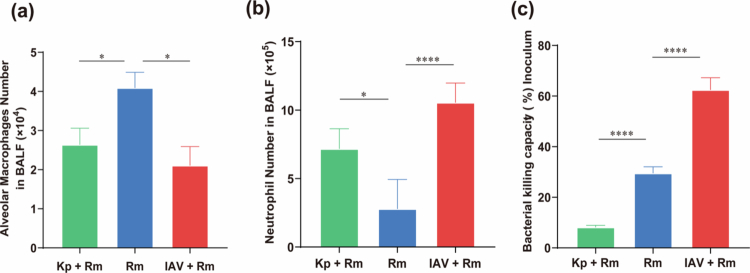
Cellular responses and functional assessment of neutrophils during pathogen co-infection. (a) and (b) Numbers of alveolar macrophages and neutrophils in BALF 12 hours post-infection of ICR mice with *R. mucilaginosa* or in combination with IAV and *K. pneumoniae* (*n* = 9–11 per group). (c) Phagocytic killing activity at 12 hours post-infection measured by CFU counts with *R. mucilaginosa* (*n* = 6–7 per group). Data are pooled from independent experiments and shown as mean ± SEM, Mann–Whitney U test; **p < 0.01, ****p < 0.0001.

**Figure 8.**
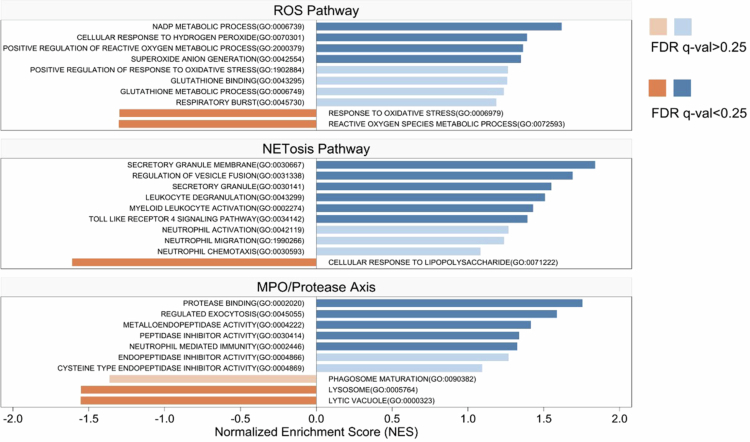
Comparative GSEA of pathways involved in neutrophil bactericidal activity. The plot displays the top 10 enriched terms within the main categories related to neutrophil bactericidal mechanisms in *K. pneumoniae* and *R. mucilaginosa* co-infection compared to IAV and *R. mucilaginosa* co-infection, including the the ROS (reactive oxygen species) pathway, the NETosis (neutrophil extracellular trap formation) pathway, and the MPO/protease (myeloperoxidase/protease) axis. They are ranked by absolute normalised enrichment score (NES). Pathways with false discovery rate (FDR)-adjusted q value < 0.25 were considered significant and are shown in dark colours; those with FDR q ≥ 0.25 are shown in light colours. *n* = 3–4 biological replicates per group.

To further dissect the underlying mechanisms, we assessed alveolar macrophage and neutrophil responses, including cell numbers and killing function, at 12 hour post-challenge (Figure 7a–c). The number of alveolar macrophages in the *R. mucilaginosa* mono-infection group was 1.5-fold higher than that in the *K. pneumoniae* and *R. mucilaginosa* co-infection group (*p* = 0.029), and 1.7-fold higher than in the IAV and *R. mucilaginosa* co-infection group (*p* = 0.018; Figure 7a). In contrast, neutrophil counts were significantly increased in both co-infections, being 2.6-fold higher in *K. pneumoniae* and *R. mucilaginosa* (*p* = 0.017) and 3.8-fold higher in IAV and *R. mucilaginosa* (*p* < 0.0001; Figure 7b) compared with mono-infection, indicating a consistent trend of enhanced recruitment. Phagocyte killing function, however, was bidirectionally modulated. It was enhanced 3.6-fold by *K. pneumoniae* pre-exposure but reduced 2.1-fold by IAV co-infection (both *p* < 0.0001; Figure 7c). To further examine the *in vivo*-associated functional state of neutrophils, we performed GSEA based on lung neutrophils transcriptome (Figure 8). Compared with the IAV and *R. mucilaginosa* co-infection group, the *K. pneumoniae* and *R. mucilaginosa* co-infection group showed broader enrichment of pathways associated with main antimicrobial functions, including oxidative stress-related responses, extracellular trap/granule programs, and protease-associated activity.

## Discussion

In this study, we developed and validated a multiplex qPCR panel for species-specific detection of *Rothia*, and further applied it in a cohort of hospitalised patients with and without respiratory infection. The results demonstrated overall high detection rates of *Rothia* spp., in particular in healthy community controls, for hospitalised patients, *Rothia* is more prevalent in asymptomatic controls and pneumonia patients but is observed less frequently in symptomatic individuals without pneumonia. This pattern suggests that its abundance reflects the immune environment shaped by the pre-existing microbiota, rather than serving as a marker of infection severity, as indicated in previous studies [29]. The asymptomatic control group may represent a relatively stable respiratory condition within the inpatient setting. By contrast, the non-pneumonia case group with respiratory symptoms often maintained an earlier or milder inflammatory state that was less favourable for *Rothia* colonisation. In patients with pneumonia, more substantial local inflammation, epithelial barrier disruption, or airway microecological remodelling may have contributed to a higher detection rate of *Rothia*. *Rothia* was a common coloniser whose clinical relevance depends on host and disease context. Our developed qPCR-based assay enables rapid and accurate detection and speciation of *Rothia*, providing strong support for elucidating the role of *Rothia* in disease progression.

TAC has been widely applied to molecular diagnostics as a syndromic approach and was adapted here for high-quality sputum, a valuable lower respiratory tract specimen with a complex composition and diverse microbiota [30]. By leveraging this specimen type, we used TAC to enable simultaneous detection of commensal *Rothia* and respiratory pathogens, define their clinical associations, provide a scalable framework for studying pathogen-pathogen interactions in respiratory diseases and clarifying the impact of co-occurrence on disease outcomes in clinical practice.

Numerous studies have investigated the causal link between oral microbiota and the development of respiratory diseases [31]. As an oral commensal, *Rothia* showed its own characteristic distribution with potential implications for disease. Respiratory infections accompanied by inflammatory responses may alter *Rothia* colonisation in the respiratory tract. The higher detection rate of *Rothia* in the pneumonia subgroup, together with the significant association observed between *R. mucilaginosa* and IAV during the influenza season, suggests that the transition from a commensal to a disease-associated state may be regulated by polymicrobial interactions. This context-dependency is supported by prior studies: its enrichment in tuberculosis coinciding with airway inflammation [32], its anti-inflammatory effects of *R. mucilaginosa* in chronic lung disease [33]. Together, these findings support a context-dependent role of *Rothia*, both as a potential protector and a contributor to disease.

Our results confirm *Rothia* species demonstrate significant positive correlations with viral pathogens including SARS-CoV-2 and IAV, and higher bacterial loads upon co-occurrence, suggesting potential synergistic effects that may exacerbate respiratory disease severity. Pathogen co-infection, especially bacterial and viral co-infection, requires heightened vigilance towards the disease. Conversely, a distinct negative association was observed for the first time with respiratory bacteria, in particular *K. pneumoniae* and *P. aeruginosa*, implying potential competitive microbial interference in the respiratory niche. Interestingly, recent work suggested pre-exposure to *R. mucilaginosa* reduced IAV burden and inflammation [34], indicating prior *Rothia* colonisation may enhance host antiviral immunity and shape the sequence of pathogen exposure alters the outcome. Further investigation of pathogen-specific time course is needed to elucidate the molecular basis of this order-dependent interplay. Taken together, respiratory microbes form dynamic networks where context determines the outcome [35,36]. Studies have shown that the detection of *Rothia* on surfaces in living environments of COVID patients was positively correlated with SARS-CoV-2 viral load, suggesting its potential as an auxiliary indicator for disease status [7]. The detection of *Rothia* can be easily incorporated into any clinical setting using qPCR platform. However, its clinical utility still requires further validation through accumulating clinical data.

Respiratory pathogens mostly demonstrated seasonality [37]. Focusing on one particular pathogen during its peak season may provide stronger analysis power. Several studies revealed the association of *Rothia* with SARS-CoV-2 during COVID pandemic [38]. Based on our two-year research period, the overall detection rates of influenza in patients with respiratory symptoms (10.1%) and those with pneumonia (10.7%) were low, resulting in its positive association with *Rothia* without statistical significance. Since the influenza has strong seasonality, we focused on the influenza season, with a much higher influenza detection rate (45.7%) ensuring the reliability of the analysis. Additionally, analysis focused on the narrow time window of influenza season is less likely to be confounded by other respiratory pathogens.

To explain the divergent outcomes, we probed the underlying cellular immune responses. Despite a concordant increase in neutrophils and decrease in macrophages across both co-infection models, their functional outcomes were opposed. Pre-exposure to IAV resulted in the recruitment of neutrophils but functional impairment, failing to control *R. mucilaginosa*, aligning with the notion that IAV impairs host defence by disrupting epithelial integrity, mucociliary clearance, and immune signalling, which promotes secondary infection [39,40]. In contrast, pre-exposure to *K. pneumoniae* enhanced host immune response, making neutrophils more effective in limiting bacterial growth [41]. These differences may reflect pathogen-specific transcriptional programming of neutrophils, leading to distinct antimicrobial capacities under different co-infection contexts. In our study, the 10^7^ CFU inoculum of *R. mucilaginosa* was chosen to establish a reproducible acute infection model. This dose was intended as a pathologically relevant challenge to animals and should not be directly extrapolated to natural colonisation conditions in healthy airways, which required further characterisation.

This study has several limitations. Although the strongest clinical association was observed between *Rothia* and SARS-CoV-2, the animal experiments were performed using an influenza virus model. Future studies using SARS-CoV-2 co-infection models are needed to directly validate this association. The single-centre design, conducted in a hospital setting among older, critically ill patients who commonly received empirical antibiotic therapy, may limit generalisability to younger, healthier populations. In addition, antibiotic exposure was highly prevalent and clinically heterogeneous in this cohort, which precluded its inclusion as a covariate in the multivariate analysis and represents an additional limitation of this study. While sputum samples offer practical utility, they may not fully represent the deep respiratory tract microbiome. We acknowledge that positive *Rothia* DNA in sputum cannot distinguish active infection, colonisation or contamination; therefore, the clinical findings represent microbial associations rather than confirmed co-infection. Moreover, as detailed clinical outcomes were not systematically analysed, the prognostic significance of *Rothia* detection remains unclear. Finally, the inferred ecological associations and underlying molecular mechanisms of *Rothia*-pathogen interactions require further validation. Future studies across multiple centres and diverse populations with various sample types are needed to refine these findings and explore the underlying mechanisms.

## Conclusions

Commensal *Rothia* species showed context-dependent association, being positively correlated with viral infections such as IAV and SARS-CoV-2 but negatively correlated with bacterial competitors like *K. pneumoniae* and *S. aureus*. Together with complementary insights from murine co-infection models, these findings highlight pathogen-specific reprogramming of innate immune function in determining the disease associations of *Rothia*, with implications for understanding pathogen interactions, diagnostics, and future interventions.

## Supplementary Material

SupplementaryMaterials16Mar2026.pdf SupplementaryMaterials16Mar2026.pdf

## Data Availability

Most data collected and analysed in this study are included within the study manuscript or in the supplemental materials. The transcriptome sequencing data generated in this study have been deposited in the NCBI Sequence Read Archive (SRA) under BioProject accession number PRJNA1437510. The remaining datasets can be made available upon reasonable request to the corresponding author after publication. This study was approved by the ethics committees of Qingdao Medical College of Qingdao University (QDU-HEC-2024246) and the Laboratory Animal Centre of Qingdao University (20241206ICR10520250819206).
