# Supplementary material for: Epidemiological characteristics and co-occurrence patterns of Rothia species and respiratory pathogens: from population surveillance to mechanistic insights
Source: J Oral Microbiol. 2026 Apr 14;18(1):2657114. doi: 10.1080/20002297.2026.2657114 (PMC13081337; doi:10.1080/20002297.2026.2657114)
Supplement: SupplementaryMaterials16Mar2026.pdf [file ZJOM_A_2657114_SM6011.pdf]

Supplementary Materials

Table of contents

Figure S1. Flowchart of patient enrollment and classification…………………….. 2

Table S1. Clinical isolates used for PCR validation and multiplex qPCR assays.…. 3

Table S2. Primer sequences for long amplicons used for qPCR confirmation.…….. 4

Figure S2. Configuration of the TaqMan Array Card for respiratory pathogens detection.…………………….………………….……………….…………….….. 5

Table S3. Baseline characteristics of community participants without respiratory symptoms and detection rates of *Rothia* species in sputum samples. … 6

Table S4. Prevalence of pathogens among patients stratified by respiratory symptoms and pneumonia status during influenza season……….………. 7

Table S5. Associations between *Rothia* species and respiratory pathogens.……….. 8

Figure S3. Survival curves of mice following intranasal infection.………………... 10

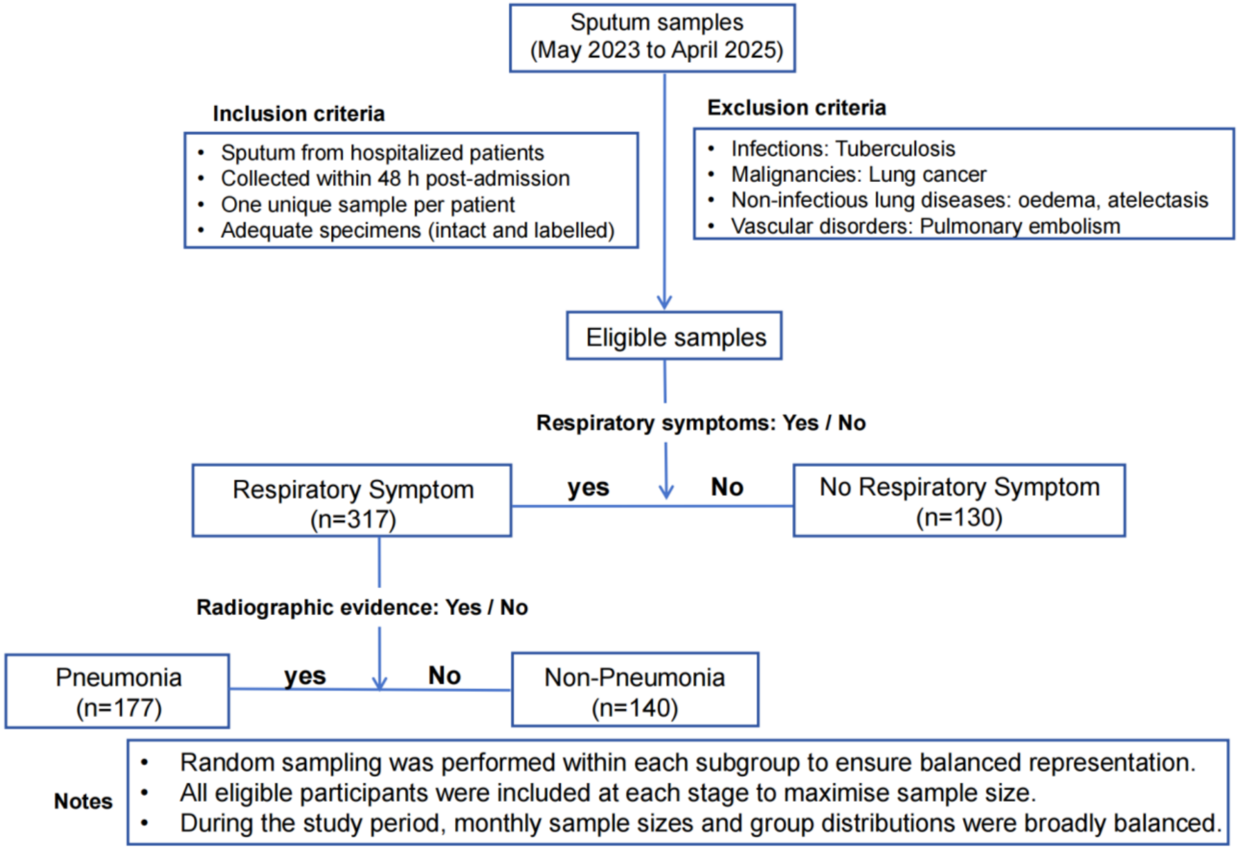

Figure S1. Flowchart of patient enrollment and classification.

Eligible sputum specimens were included, and patients were classified according to respiratory symptoms and pneumonia status.

Table S1. Clinical isolates used for PCR validation and multiplex qPCR assays.

**Species**	**No. of isolates (n=77)**
Acinetobacter baumannii	6
Aeromonas hydrophila	2
Enterobacter cloacae complex	8
Enterococcus faecalis	2
Escherichia coli	13
Klebsiella aerogenes	1
Klebsiella pneumoniae	10
Moraxe catarrhalis	1
Nocardia spp.	2
Proteus mirabilis	4
Proteus penneri	1
Proteus vulgaris	1
Providencia rettgeri	2
Pseudomonas aeruginosa	7
Pseudomonas stutzeri	1
Serratia marcescens	2
Staphylococcus aureus	3
Staphylococcus epidermidis	5
Staphylococcus haemolyticus	3
Staphylococcus simulans	1
Staphylococcus warneri	1
Streptococcus agalactiae	2

The table lists the bacterial species isolated from clinical samples and their corresponding strain counts, which were used to validate the specificity of the *Rothia* detection method. No amplification was observed for these bacteria, indicating that the method accurately distinguishes *Rothia* from a broad range of other bacterial pathogens.

Table S2. Primer sequences for long amplicons used for qPCR confirmation.

Pathogen	Primer	Sequence	Accuracy
R. aeria	F	AGCTGCCTGAGCGTATCAAT	100%（20/20）
	R	CAATACGCCCAATGAACATG	
R. dentocariosa	F	CCTGATGAGGATGCTCTCCTT	100%（20/20）
	R	GATAGGAATGATAGCGGCGG	
R. mucilaginosa	F	TATTCGAATTGTGGCAGTCG	100%（34/34）
	R	TGAACCTGGCGAACATTGT	

This table lists the long-amplicon primers for PCR detection of *R. aeria*, *R. dentocariosa*, and *R. mucilaginosa*, with forward (F) and reverse (R) sequences, and presents the clinical specificity results. The assay achieved 100% positive rate and accuracy, demonstrating high specificity of the newly developed qPCR method.

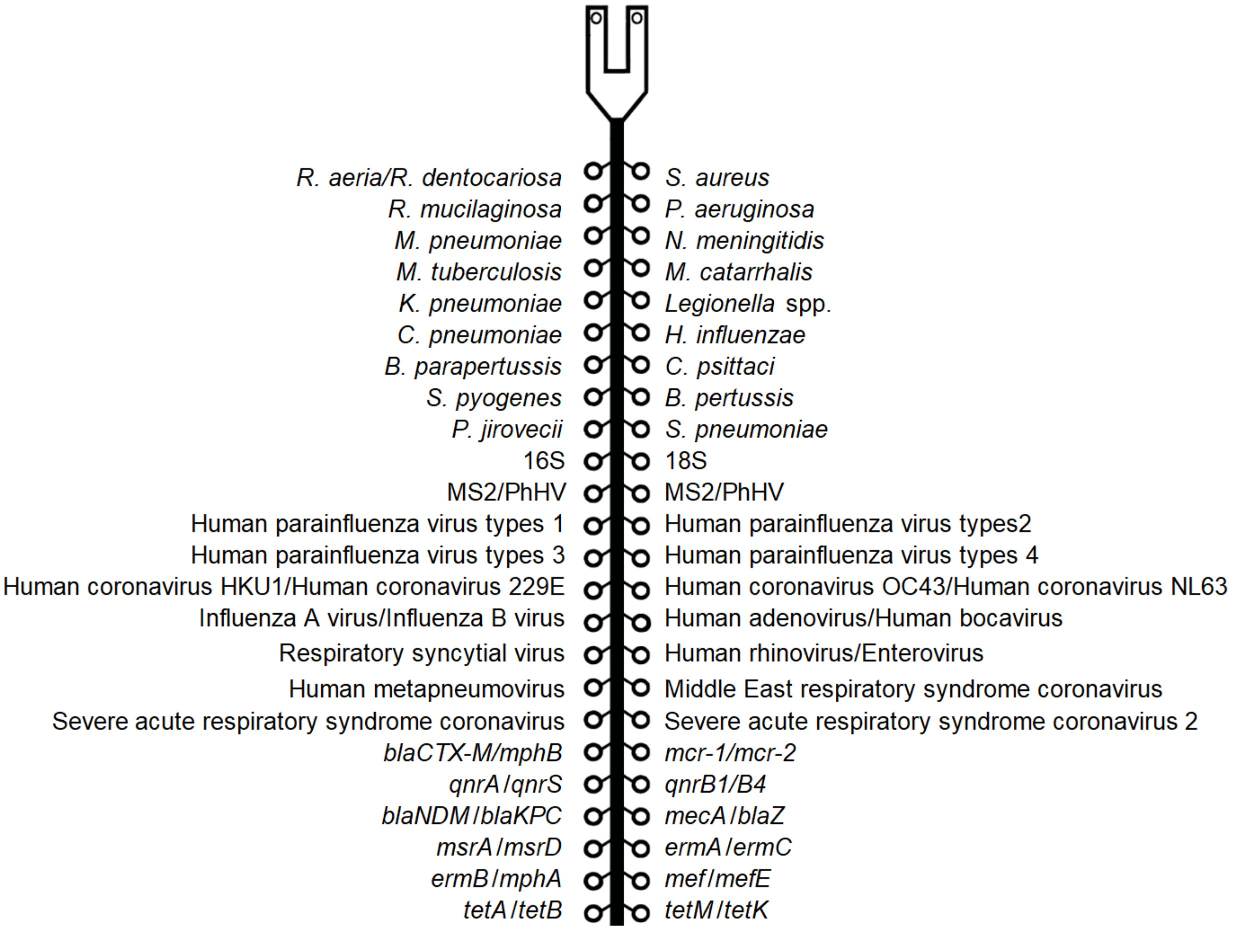

Figure S2. Configuration of the TaqMan Array Card for respiratory pathogens detection.

Table S3. Baseline characteristics of community participants without respiratory symptoms and detection rates of *Rothia* species in sputum samples.

**Variables**	**All participants (N = 84)**
Age, years	71 (64.8–75.3)
Sex	
Male	54 (64.3%)
Female	30 (35.7%)
BMI, kg/m²	25.4 (23.8–27.3)
Comorbidities	
Diabetes mellitus	12 (14.1%)
Hypertension	35 (41.2%)
Atherosclerosis	4 (4.7%)
Heart failure	12 (14.1%)
Cerebral infarction	1 (1.2%)
No Comorbidities	42 (50%)
Detection of *Rothia* species	
R. aeria	71 (84.5%)
R. dentocariosa	72 (85.7%)
R. mucilaginosa	75 (89.3%)
No *Rothia* species detected	4 (4.8%)

Data are presented as median (IQR) or n (%). Information was obtained by questionnaire. All participants had no respiratory symptom and had not taken any antibiotics within 1 month before sample collection.

Table S4. Prevalence of pathogens among patients stratified by respiratory symptoms and pneumonia status during influenza season.

Pathogen	Respiratory Symptoms (n=65)	Respiratory Symptoms	
		Non-Pneumonia (n=30)	Pneumonia (n=35)
Bacteria			
H. influenzae†	9 (13.8)	4 (13.3)	5 (14.3)
K. pneumoniae†	12 (18.5)	6 (20.0)	6 (17.1)
S. aureus†	11 (16.9)	4 (13.3)	7 (20.0)
M. pneumoniae†	7 (10.8)	1 (3.3)	6 (17.1)
S. pneumoniae	6 (9.2)	1 (3.3)	5 (14.3)
M. catarrhalis	6 (9.2)	4 (13.3)	2 (5.7)
P. aeruginosa	5 (7.7)	2 (6.7)	3 (8.6)
Virus			
Influenza A virus†	21 (32.3)	5 (16.7)	16 (45.7)
Human rhinovirus	3 (4.6)	2 (6.7)	1 (2.9)
SARS-CoV-2	3 (4.6)	1 (3.3)	2 (5.7)
Human metapneumovirus	3 (4.6)	1 (3.3)	2 (5.7)

Pathogens with a detection rate >10% in the respiratory symptom group were defined as key pathogens and denoted by † in tables.

Table S5. Associations between *Rothia* species and respiratory pathogens. The odds ratios were shown with 95% confidence interval.

(a) Associations of *Rothia* species with respiratory pathogens from May 2023 to April 2025.

Respiratory infection (n=317)

	IAV	SARS-CoV-2	*H. influenzae*	*P. aeruginosa*	*S. aureus*	*K. pneumoniae*
R. aeria	1.4 (0.7–2.9)	2.2 (1.3–3.7)	2.8 (1.3–5.8)	1.0 (0.5–1.8)	1.2 (0.7–2.2)	0.4 (0.2–0.7)
R. dentocariosa	2.2 (1.0–4.8)	2.0 (1.2–3.5)	4.0 (1.8–9.2)	0.5 (0.3–1.0)	1.9 (1.0–3.4)	0.4 (0.2–0.7)
R. mucilaginosa	1.8 (0.8–3.9)	4.0 (2.1–7.5)	2.3 (1.0–5.1)	0.4 (0.2–0.8)	0.7 (0.4–1.3)	0.3 (0.2–0.5)
Any *Rothia*	3.7 (1.3–10.9)	4.2 (2.0–8.8)	2.4 (1.0–6.1)	0.4 (0.2–0.7)	1.3 (0.7–2.6)	0.3 (0.2–0.5)

Pneumonia (n=177)

	IAV	SARS-CoV-2	*H. influenzae*	*P. aeruginosa*	*S. aureus*	*K. pneumoniae*
R. aeria	1.6 (0.6–4.0)	1.4 (0.7–2.7)	2.0 (0.8–5.2)	0.8 (0.3–1.9)	0.9 (0.4–2.1)	0.5 (0.2–1.0)
R. dentocariosa	1.6 (0.6–4.4)	1.4 (0.7–2.7)	3.8 (1.2–12.1)	0.6 (0.2–1.4)	1.4 (0.6–3.4)	0.4 (0.2–0.9)
R. mucilaginosa	2.0 (0.6–6.3)	2.7 (1.2–6.2)	1.4 (0.5–4.2)	0.4 (0.2–1.1)	0.6 (0.2–1.4)	0.3 (0.1–0.6)
Any *Rothia*	3.1 (0.7–13.9)	2.4 (0.9–6.2)	1.9 (0.5–6.8)	0.4 (0.2–1.0)	1.1 (0.4–2.8)	0.3 (0.1–0.6)

(b) Associations of *Rothia* species with respiratory pathogens during influenza season from December 2024 to February 2025.

Pneumonia (n=35)

Rothia detection rate (%)		IAV	*M. pneumoniae*	*H. influenzae*	*S. aureus*	*K. pneumoniae*
R. aeria	12 (34.3)	2.2 (0.5–9.0)	5.2 (0.8–34.5)	3.5 (0.5–24.6)	0.7 (0.1–4.4)	0.3 (0.0–3.2)
R. dentocariosa	17 (48.6)	1.9 (0.5–7.2)	1.9 (0.3–11.8)	4.0 (0.4–40.1)	2.5 (0.4–15.1)	0.8 (0.1–4.7)
R. mucilaginosa	19 (54.3)	8.4 (1.8–38.6)	7.1 (0.7–68.6)	0.7 (0.1–4.6)	0.1 (0.0–1.2)	0.4 (0.3–0.6)
Any *Rothia*	23 (65.7)	7.8 (1.4–44.0)	3.1 (0.3–29.7)	2.3 (0.2–23.4)	1.4 (0.2–8.5)	0.5 (0.1–2.7)

Figure S3. Survival curves of mice following intranasal infection.

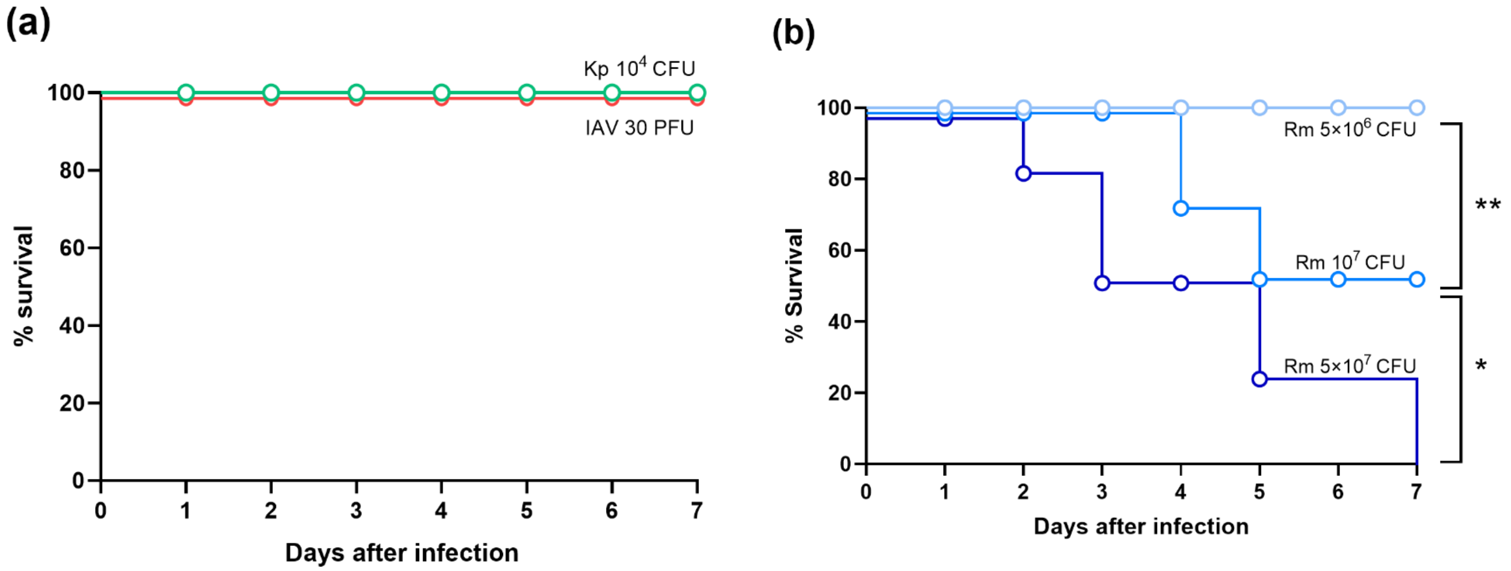

(a) Survival of ICR mice after infection with *K. pneumoniae* (10^4 CFU) or IAV (30 PFU). (n=7 per group).

(b) Survival of ICR mice after infection with increasing doses of *R. mucilaginosa* (5×10^6, 10^7, or 5×10^7 CFU) ($n = 7–14$ per group).

Survival curves were analysed by log-rank (Mantel–Cox) test with Bonferroni correction. *$p<0.05$, **$p<0.01$.
